# External Knowledge Search and Innovation Performance in Iranian Biopharmaceutical Firms: The Mediating Role of Knowledge Integration Capability

**DOI:** 10.5812/ijpr-164658

**Published:** 2026-05-15

**Authors:** Fatemeh Babaei, Nazila Yousefi, Mohammad Peikanpour

**Affiliations:** 1 Ph.D. candidate of Pharmacoeconomics and Pharma Management, School of Pharmacy, Shahid Beheshti University of Medical Sciences, Iran; 2 Associate Professor in Pharmacoeconomics and Pharma Management, School of Pharmacy, Shahid Beheshti University of Medical Sciences, Iran; 3 Assistant Professor of Pharmacoeconomics and Pharma Management, School of Pharmacy, Shahid Beheshti University of Medical Sciences, Iran

**Keywords:** Innovation Performance, R&D Intensity, External knowledge search strategy, Pharmaceutical Industry, Biopharmaceutical, Knowledge Integration Capability

## Abstract

**Background:**

In the biopharmaceutical sector, characterized by rapid technological change and intense market competition, external knowledge search (EKS) has become a strategic necessity to overcome the limitations of closed innovation. However, while EKS is widely studied in developed economies, its underlying mechanisms remain underexplored in emerging biopharmaceutical sectors.

**Objectives:**

This cross-sectional study examines how EKS affects innovation performance (IP) in Iranian biopharmaceutical firms, testing the mediating role of knowledge integration capability (KIC) and the moderating effect of R&D intensity, while controlling for firm size and age.

**Methods:**

Data were collected using a validated questionnaire completed by 92 senior executives (CEOs, R&D, and Business Development managers) from 44 Iranian biopharmaceutical companies. The model was analyzed using partial least squares structural equation modeling (PLS-SEM) in SmartPLS.

**Results:**

EKS significantly enhances both IP (β = 0.462, P < 0.001) and KIC (β = 0.239, P < 0.003). KIC positively influences IP (β = 0.437, P < 0.001) and partially mediates the EKS-IP relationship (indirect effect β = 0.104, P = 0.02). R&D intensity showed no significant moderating effect (β = 0.198, P = 0.10). The model explains 40.5% of IP variance (R² = 0.405). Firm size (β = 0.009, P = 0.924) and age (β = 0.030, P = 0.792) had no significant effects.

**Conclusions:**

The impact of EKS on IP depends more on a firm’s KIC than on its R&D intensity. This study advances open innovation theory by clarifying how external knowledge is effectively translated into innovation outcomes within an emerging biopharmaceutical context.

## 1. Background

Innovation is essential for gaining a competitive edge and ensuring long-term success. To foster innovation, companies need to move beyond their internal resources and seek out external information, skills, and capabilities (1, 2). As organizations manage the challenges of technological progress and market changes, strategically seeking out and integrating external knowledge has become crucial for driving innovation (3, 4). EKS strategies comprise various activities, including scouting for cutting-edge technologies, engaging with industry experts, forming alliances with academic institutions, and participating in open innovation platforms. These strategies help companies gain and integrate a wide range of knowledge, which is essential for creating new and innovative products, processes, and services. The effect of external knowledge on company innovation is substantial, and companies that actively pursue new knowledge sources often show higher levels of innovation (5, 6).

Historically, the pharmaceutical industry relied on closed innovation, protecting its new discoveries through patents, giving manufacturers exclusive intellectual property rights (IPR). This strategy primarily focused on protecting the company's proprietary knowledge and minimizing external knowledge exchange (7). However, rising R&D costs (now exceeding $2.6 billion per drug), regulatory hurdles, and pressure to accelerate drug development have made this model unsustainable (8-10). Given the many challenges in the political, regulatory, and economic landscape of today, pharmaceutical companies are under pressure to speed up product development, manage costs, enhance design processes, prioritize areas with high medical requirements, and significantly boost productivity without compromising quality. As a result, the sector has increasingly embraced open innovation, forming partnerships with academia, biotech firms, and even competitors to enhance R&D efficiency and speed up patient access to new therapies (11-13).

Although the entire pharmaceutical industry faces innovation challenges, biopharmaceutical companies face even greater ones. Developing biologic drugs is far more complex than traditional medicines. They involve longer R&D cycles, stricter regulations, and a much stronger need for external scientific knowledge (14, 15). In contexts such as Iran, where companies work under specific institutional and resource limitations, relying only on internal R&D is often not enough. This highlights the need to understand how firms can effectively translate external knowledge into innovation outcomes. To address this, our study examines two key mechanisms: first, whether KIC enables the conversion of external knowledge into IP, and second, whether R&D intensity strengthens this relationship.

### 1.1. Conceptual Framework and Hypotheses

This research is based on a framework grounded in the principles of knowledge-based view (KBV) theory. The theory emphasizes that knowledge is the most important strategic resource for an organization. It stresses the significance of continuous learning and innovation, suggesting that firms that can learn and adapt quickly are better equipped to innovate and respond to market changes. The theory underscores the need for firms to effectively manage and integrate knowledge to maintain a competitive advantage in the modern economy. This involves processes for acquiring, storing, sharing, and using knowledge to achieve organizational goals (16-18). Finally, this article enhances the understanding of the knowledge-based theory by emphasizing the significance of knowledge as a strategic resource and its effect on IP.

### 1.1.1. External Knowledge Search Strategy

The concept of External knowledge search strategy is crucial in strategic management and innovation literature. It refers to the structured approach companies use to identify, acquire, and leverage knowledge from external sources to boost their innovation potential and remain competitive. This strategy is grounded in the knowledge-based perspective of the firm (6).

In this study, we assessed the EKS strategy through external knowledge search depth. We intend to conduct our evaluation in the biopharmaceutical industry where knowledge depth represents a vertical dimension that reflects the level of collaboration with partners. It also signifies the complexity, uniqueness, and knowledge content within the field (19-21).

### 1.1.2. Knowledge Integration Capability

A knowledge base by itself may not be enough for effective product development. A company also needs to implement knowledge integration mechanisms to gather, understand, and apply its knowledge resources (22). KIC is the ability of an organization to evaluate and combine knowledge from external sources and its own internal experiences. This ability is essential for organizational learning and the decision-making process, as it allows the organization to create knowledge, which is then retained and integrated into its practices and procedures (23). Ming-Chao Wang defines KIC as a firm's capability to integrate and utilize knowledge and expertise from its operational processes, suppliers, and partners (24). In a different study, KIC was described as a project-oriented firm's capability to intentionally generate new knowledge by combining various knowledge resources, expand this knowledge to activities that create value, and adapt it to meet evolving market conditions (25). Ming-Shun Li describes KIC as the ability to leverage existing knowledge to create new reconfigurations and combinations, which act as a foundation for innovation (26).

The concept of KIC has been interpreted in various ways by scholars from different perspectives. However, the fundamental understanding remains consistent. In essence, this process involves collecting distinct types of knowledge, then reorganizing and innovating based on the members' comprehension and assimilation. The aim is to preserve the organization's core competitiveness.

### 1.1.3. Innovation Performance

Innovation performance (IP) refers to the ability of companies to use new ideas and technologies to surpass their competitors and gain a competitive edge. It involves assessing an organization's capacity to create and apply innovations, measuring the impact of these efforts on actual innovative outcomes, and determining how they have contributed to the organization's success and expansion (27).

IP can be assessed by the number of sales resulting from new or improved technological products (28), number of new product development and patents (29-31), customer value creation (32), the success ratio of new services or products, cost reductions (31), and access to new markets (30). In essence, IP is a multidimensional concept that measures how effectively organizations can convert their innovation efforts into concrete outcomes that contribute to their strategic goals and market success (33). It is a measure of how effectively an organization can utilize its innovative capabilities to achieve its goals, which was the focus of our study.

### 1.1.4. External Knowledge Search Strategy and Innovation Performance

IP is crucial for a company's competitive edge and long-term prosperity. Effective innovation relies on various factors, including the strategies used to obtain and apply knowledge. One crucial strategy is EKS, which involves organizations seeking valuable new ideas from external sources such as public and private institutions, competitors, suppliers, and customers. This approach is vital for fostering innovation as it enables firms to access diverse perspectives, new technologies, and market trends that may not be available internally (20). In addition, companies increasingly rely on external knowledge resources to maintain a sustainable competitive advantage in today's markets (6). Recent research has emphasized the beneficial effects of EKS on IP. Segarra-Ciprés and Bou-Llusar (2018) discovered that firms conducting extensive and in-depth EKS are more likely to achieve better innovation outcomes. However, they noted that knowledge search strategies might not always yield the desired results. Therefore, firms should ensure that their search strategies are aligned with their innovation goals and the specific contexts of their industry (34). Zhang et al. (2022) (5) conducted a meta-analysis and concluded that EKS positively correlates with firm IP, particularly in mature enterprises. Wang et al. (2020) highlight the importance of an ambidextrous knowledge search strategy, which seeks to balance the depth and breadth of external knowledge. This strategy enables firms to effectively integrate a broad array of external knowledge sources. As a result, this integration leads to improved innovation performance (35). Recent scientific articles provide strong evidence that searching for external knowledge is a crucial factor for driving IP. The capability to effectively find and incorporate external knowledge into a company's innovation processes is essential for staying competitive in a fast-changing business environment. These studies offer valuable insights into how companies can strategically manage their knowledge search activities to maximize innovation outcomes. Based on the theoretical foundations and empirical evidence, we propose the following hypothesis:

H1: External knowledge search strategy will exert a positive and significant effect on companies’ innovation performance.

### 1.1.5. External Knowledge Search Strategy, Knowledge Integration Capability, and Innovation Performance

Acquiring external knowledge is essential for organizations to secure a competitive edge and drive product innovation (36, 37). The enterprise should integrate that new external knowledge with existing ones through a knowledge integration mechanism to create innovative knowledge resources (24). Knowledge integration is the ability to efficiently handle and apply both newly acquired and existing knowledge within organizations. It enables enterprises to obtain, distribute, and apply knowledge (6, 38). Integrating knowledge allows businesses to enhance their existing repository of knowledge, speed up the generation of new knowledge, and offer valuable insights for pioneering innovations (39).

Recent research underscores the significance of aligning EKS strategies with the capability to effectively integrate that knowledge. For instance, a study by Zhang, Wang, and Xu (2021) emphasizes that knowledge integration serves as a crucial intermediary between breakthrough innovation and knowledge search in enterprises (39). This implies that simply obtaining external knowledge is not sufficient; to achieve innovation outcomes, companies must also possess the capability to effectively combine this knowledge. Liu's study suggests that the KIC mediates the impact of external search strategies on both incremental and radical IP. This mediation effect underscores the importance of KIC as a critical factor in turning external knowledge into innovative outcomes (40). The literature suggests that taking a strategic approach to seeking external knowledge, along with having a strong ability to integrate that knowledge, is crucial for promoting innovation. Organizations need to concentrate not only on obtaining external knowledge but also on creating the necessary processes and systems to integrate this knowledge effectively. As the business environment continues to change, the capability to integrate external knowledge will continue to be a critical factor for achieving innovation success and organizational sustainability (25).

The review above draws on the latest articles and summarizes the current understanding of the relationship between EKS strategies, KIC, and IP. Based on this, the following hypothesis is proposed: Organizations that actively seek knowledge beyond their borders are more likely to develop advanced capabilities in integrating diverse knowledge sources.

H2: External knowledge search strategy will exert a positive and significant effect on knowledge integration capability.

H3: Knowledge integration capability will exert a positive and significant effect on innovation performance.

Ultimately, the effectiveness of an EKS strategy on IP hinges on the organization's ability to integrate and effectively utilize the acquired knowledge.

H4: External knowledge search strategy will exert a positive and significant effect on innovation performance with the mediating effect of knowledge integration capability.

### 1.1.6. The Moderating Effect of R&D Intensity on Innovation Performance

IP refers to the results of a company's innovative activities, including the development of new products, the speed of innovation, and the economic gains from these innovations (31). Usai et al. (2021) emphasize that a firm's success is significantly measured by its ability to transform R&D efforts into marketable solutions (41).

Xu, Wang, and Liu (2021) identified a strong positive correlation between IP and R&D investment (42). Similarly, Nooteboom et al. (2007) observed that higher R&D intensity correlates with improved IP, leading to more patents for firms already proficient in this area (43). Zhu et al. (2019) demonstrated that R&D personnel input and R&D investment significantly enhance technological IP in most high-technology based industries in China (44).

A study contends that external knowledge acquisition and internal R&D are complementary activities in the innovation process. The extent of this complementarity is influenced by factors such as the firm's R&D intensity (45). This implies that companies with higher R&D intensity may be better positioned to benefit from EKS strategies. Therefore, we propose the following hypothesis:

H5: External knowledge search strategy will exert a positive and significant effect on innovation performance with the moderating effect of R&D intensity.

### 1.1.7. Firm Size and Age as Control Variables on the Model

A company's size and age can influence various aspects of its operations. Larger firms typically have more resources to invest in R&D, which can result in greater innovation. A study suggests that larger firms can financially benefit from environmental innovation, especially when driven by regulations or industry codes of conduct (46). Another study discovered that the size of a firm moderates the impact of innovation on its performance, Therefore, firms should consider their size before investing in innovative projects or process management improvements, as it can influence how creativity is perceived within the organization and its overall performance (47).

Another factor that has been linked to IP is firm age. Coad et al. review the literature on this topic, suggesting that older firms might benefit from greater experience and established processes that support innovation. However, the link between a firm's age and its innovation is intricate and not easily defined (48). Some studies suggest that established routines and resistance to change in older firms may lead to less inclination to innovate (49, 50). Conversely, some research suggests that the age of a firm can have a positive impact on innovation by offering a stable foundation for continuous improvement (51).

The literature review indicates that both firm size and age have intricate relationships with IP. Larger firms have more resources for innovation, but the effectiveness of these innovations can be affected by external factors like market demands and regulatory pressures. Similarly, older firms may have more experience, but their established routines can either hinder or facilitate innovation. Drawing from the literature review, we propose the following hypotheses:

H6: Firm size will exert a positive and significant effect on the company's innovation performance.

H7: Firm age will exert a positive and significant effect on the company's innovation performance.

Building on the KBV introduced earlier, our hypotheses are explicitly designed to reflect its core tenets. Specifically, the relationship between EKS and IP (H1) embodies the KBV principle that access to external knowledge is a strategic imperative. The positive effect of EKS on KIC (H2), and of KIC on IP (H3), operationalize the theory’s central claim that value is created not merely by acquiring knowledge, but by effectively internalizing and recombining it. The mediating role of KIC (H4) further captures the KBV insight that competitive advantage stems from how firms process knowledge, not just its collection. Finally, the test of R&D intensity as a moderator (H5) allows us to examine whether structural resources alone can compensate for limited integrative capability. Together, these hypotheses translate the abstract logic of KBV into a testable model of innovation in a resource-constrained biopharmaceutical context.

**Figure 1.**
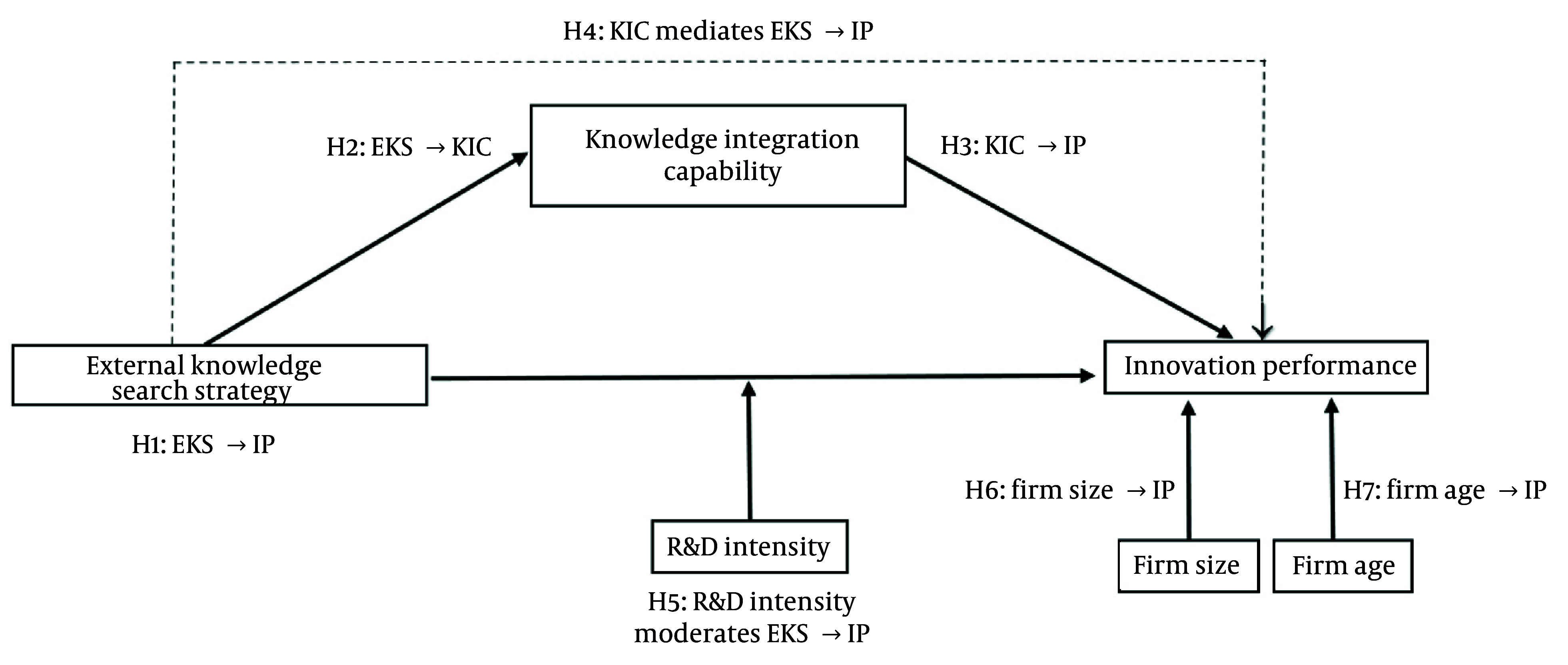
Conceptual framework of the study model

Figure 1 illustrates the concepts and connections proposed in the hypotheses. The model illustrates EKS as independent variable, with IP as the dependent variable. It also includes the moderating effect of R&D intensity and the mediating effect of KIC components.

## 2. Objectives

This study examined whether KIC enables the conversion of external knowledge into IP and whether R&D intensity strengthens this relationship in Iranian biopharmaceutical firms.

## 3. Methods

### 3.1. Population and Sample

In this study, we focused on biopharmaceutical companies in Iran. The list of companies was obtained from the Iranian Food and Drug Administration (IFDA). After excluding companies that had no activity or products in the last 5 years, 47 biopharmaceutical companies were selected as the final sample. To mitigate common method bias, data were gathered from multiple respondents within each company. The questionnaire was distributed to selected companies, requesting input from 3 individuals, including the CEO, R&D manager, and business development managers. Out of 141 distributed questionnaires, 92 were completed and returned by 44 companies.

### 3.2. Instrument and Data Collection

We developed the questionnaire by adapting items from previous studies (see Section 3.3 for details) and validated it using the Content Validity Index (CVI) and Content Validity Ratio (CVR) methods. The questionnaire was separated into two sections. The first section gathers general information about the respondent, such as their job position and history of his/her activity in that company, along with the company's profile, including its name, year of establishment, total number of employees, and the number of employees in the R&D department. The second section assesses the research variables using a five- or seven-point Likert scale.

Following Armstrong and Overton's (1977) procedure, t-tests were conducted to compare nonresponding and responding firms in terms of size and age to address potential nonresponse bias. The findings revealed no significant differences, suggesting that nonresponse bias was not a concern (52).

### 3.3. Measures

#### 3.3.1. Dependent Variable

We evaluated IP using a scale created by Baker and Sinkula, Ritter and Gemünden, Kirner et al., and Zhou et al. Participants were requested to rate their agreement with different statements on a 7-point scale (53-56).

#### 3.3.2. Independent Variables

To measure EKS strategy, we relied on three studies by Laursen and Salter, Guo and Wang, and Kang (20, 57, 58). We asked our respondents which sources of external knowledge and to what extent they use for new product design. This variable was constructed by combining 17 possible sources of knowledge for information obtained from the mentioned sources: (1) Suppliers of materials, equipment, parts, or software (2) Competitors; (3) Customers; (4) Business consultants; (5) Informal communications with employees of other companies; (6) Recruiting experienced employees from other companies; (7) Consulting contract with a foreign party for the technical knowledge transfer or training of manpower; (8) Exhibitions; (9) Patents; (10) Scientific magazines; (11) Technical and commercial press or websites; (12) Specialized and international conferences; (13) National meetings and congresses; (14) Business associations such as chambers of commerce; (15) Commercial laboratories/ R&D companies; (16) Government or private research organizations; and (17) Universities or other higher education institutions. Each of the 17 sources used a 5-point Likert scale to measure the external knowledge depth.

#### 3.3.3. Mediator and Moderator Variables

KIC functioned as a mediator in our model, and we relied on three studies to measure it. All questions were measured using a 7-point Likert scale (25, 39, 59). According to Katila and Ahuja, we calculated R&D intensity (moderator variable) as a firm's R&D expenditures divided by its total sales (60).

#### 3.3.4. Control Variables

Firm size and age are commonly used control variables in innovation research, as they reflect differences in resource availability and organizational experience that may affect IP. We included these two variables because they are standard controls in studies on EKS (20)and are particularly relevant in resource-constrained settings like Iran’s biopharmaceutical sector. Other potential controls, such as market competition or regulatory pressure, were not included because all firms in the sample operate under the same national regulatory authority (IFDA) and face highly similar market conditions, resulting in limited variation that would not meaningfully contribute to the model.

Firm age was calculated as the natural logarithm of the number of years since the company's establishment, while firm size was measured by the logarithm of the number of employees (20, 61).

### 3.4. Statistical Analysis

We utilized partial least squares structural equation modeling (PLS-SEM) with Smart PLS 3.0 to validate our model and hypotheses. PLS, as a multivariate analysis method, allows researchers to simultaneously examine latent and manifest variables (62). PLS-SEM is suitable for this study for several reasons. Firstly, it can handle non-normal data (63). We performed the Kolmogorov-Smirnov test and found that aside from EKS, our other two variables (IP and KIC) were not normally distributed. Additionally, PLS-SEM can handle small sample sizes, making it suitable for our analysis as we had 92 questionnaires (64).

## 4. Results

Table 1 provides information on the age and R&D intensity of the companies. The table also shows the distribution of company size based on the number of employees, according to the OECD classification. 55% of the companies in the study were considered medium-sized, with 250 - 499 employees. Additionally, almost half of the companies had an R&D intensity between 15% and 25%.

**Table 1.** Investigation of Age, Size, and R&D Intensity in Biopharmaceutical Firms

Variables and Categories	Percentage
**Firm age (y) ** ^**a**^	
5 or less	4.5
6 - 15	50
16 - 40	36.4
More than 40	9.1
**Firm size (enterprise) ** ^**b**^	
Small	25
Medium	54.5
Large	20.5
**R&D intensity (%) ** ^**c**^	
Less than 5	7
5 - 15	20
15 - 25	52
25 - 35	14
More than 35	7

^a^ Calculated as the natural logarithm of the number of years since the company's establishment.

^b^ Calculated by the logarithm of the number of employees

^c^ Calculated as R&D expenditure/total sales × 100%, based on self-reported financial data.

### 4.1. Data Validity and Reliability

We began by conducting several tests to confirm the reliability and validity of our measurements. Initially, we demonstrated that all items were appropriately loaded onto their respective hypothetical factors, with all factor loadings exceeding 0.60 (65). We evaluated the construct's reliability, as presented in Table 2. All values for Cronbach's alpha and composite reliability (CR) exceeded 0.7, demonstrating strong measurement reliability (66). Next, we evaluated the construct validity by examining the component items of each scale to establish both convergent and discriminant validity.

To verify convergent validity, we calculated the average variance extracted (AVE). The AVE scores for all three variables surpassed the recommended threshold of 0.5, thereby confirming convergent validity (67). The research constructs were assessed for discriminant validity using the Fornell-Larcker criterion (66). The findings show that each construct's average variance extracted is significantly higher than its highest shared variance with other constructs, offering additional proof of discriminant validity (67). Therefore, it can be inferred that the measures employed in this study demonstrate adequate reliability and validity.

**Table 2.** Reliability and Validity of Constructs ^a, b^

Constructs and Items	Factor Loading	AVE	Cronbach’s α	CR
**External knowledge search strategy**		0.549	0.736	0.829
Exhibitions	0.817			
Press or technical and commercial sites	0.635			
Specialized and international conferences	0.764			
National meetings and congresses	0.737			
**Knowledge Integration Capability**		0.584	0.860	0.894
Our company can integrate the acquired EK into organizational performance.	0.782			
In our company, team meetings involve experts with different skills to generate new ideas.	0.729			
Our company shares information from experts in different units with relevant people in the organization.	0.716			
Employees in the company have a shared understanding of the processes required to complete projects.	0.781			
The company's staff is well-informed about and complies with the necessary rules, responsibilities, and collaboration measures	0.786			
All employees in the company can use their expertise and knowledge to bring new ideas to fruition.	0.789			
**Innovation performance**		0.533	0.875	0.900
Our company is seeing an increase in the introduction of new products.	0.741			
Our new product development program has met its set objectives.	0.749			
Our new products clearly offer more advantages than the previous ones.	0.712			
Our overall new product development program is much more successful compared to that of our main competitors.	0.765			
We always release new products before our competitors.	0.640			
Our new products are gaining more market share.	0.891			
Our new products create new markets for themselves.	0.701			
After our new products entered the market, they achieved remarkable success in terms of sales.	0.606			

^a^ 0.5 ≤ factor loading < 0.7, average variance extracted (AVE) ≥ 0.5, Cronbach’s α (CA) ≥ 0.7, composite reliability (CR) ≥ 0.7.

^b^ All constructs were measured using a 5- or 7-point Likert scale.

### 4.2. Structural Model Fitness

We assessed the variance inflation factors (VIFs) for all constructs in our model to detect multicollinearity. The VIFs are all below 1.5, significantly lower than the widely accepted threshold of 5 (68). We utilized the R-squared value for the dependent variables to evaluate the structural model; 0.405 for IP, and 0.206 for KIC. The structural model explained (40.5%) variance in IP and (20.6%) variance in KIC.

### 4.3. Hypothesis Testing and Results

We used bootstrapping to analyze the hypotheses and explore the significance level among all the variables. In line with our first hypothesis, the EKS strategy has a positive effect on IP (β = 0.462, P < 0.001). Our results indicate that EKS also has a significant positive effect on KIC (β = 0.239, P < 0.003), and KIC has a positive significant effect on IP (β = 0.437, P < 0.001). Consistent with H4, KIC mediates the effect of EKS on IP. We perform the Sobel test to obtain the path coefficient of the mediating variable. To determine the level of mediation, we calculated the variance accounted for (VAF) at 0.2, indicating a partial mediation effect. The VAF value of 0.2 indicates that 20% of the total effect of EKS on IP is transmitted through KIC, confirming a partial but meaningful mediating role. Meanwhile, an R² of 0.405 for IP suggests that the model explains over 40% of the variance in IP a substantial level in organizational and innovation research, particularly within the context of emerging biopharmaceutical firms facing resource constraints. In our study, we found that R&D intensity did not have a significant moderating effect on IP (β = 0.198, P = 0.10). Finally, neither firm age nor size had a significant effect on IP (Table 3).

**Table 3.** Results of Hypothesis Testing

Hypothesis	Statement	P-Value	beta	Result
**H1**	The EKS strategy will exert a positive and significant effect on the company's IP.	< 0.000 ^a^	0.462	Supported
**H2**	EKS strategy will exert a positive and significant effect on KIC.	< 0.003 ^a^	0.239	Supported
**H3**	KIC will exert a positive and significant effect on IP.	< 0.001 ^a^	0.437	Supported
**H4**	EKS strategy will exert a positive and significant effect on IP with the mediating effect of KIC.	0.02 ^a^	0.104	Supported
**H5**	EKS strategy will exert a positive and significant effect on IP with the moderating effect of R&D intensity.	0.1	0.198	Not supported
**H6**	Firm size will exert a positive and significant effect on the company's innovation performance.	0.924	0.009	Not supported
**H7**	Firm age will exert a positive and significant effect on the company's innovation performance.	0.792	0.030	Not supported

P ≤ 0.05.

## 5. Discussion

### 5.1. Confirming the Effect of External Knowledge Search Strategy on Innovation Performance

The relationship between EKS and IP has always been the focus of research in the field of knowledge management and innovation. The findings of this study confirm the positive and significant effect of EKS strategy on IP. This aligns with the results of a meta-analysis conducted by Zhang et al., which demonstrated a significant positive correlation between EKS and IP. The study suggests that companies actively seeking knowledge beyond their borders are more likely to achieve better innovation outcomes (5). Zhengrui Li's study in 2023 pointed out that seeking market information and technical knowledge to understand market needs and trends positively affects the quality and speed of innovation development (69). In our study, we found that biopharmaceutical companies are currently benefiting from 17 different sources of external knowledge, including patents, customer, supplier, and competitor knowledge, and more. Our research showed that companies can improve their IP by leveraging press, technical, and commercial knowledge sources, and by participating in specialized and international conferences, exhibitions, national meetings, and congresses.

The findings from similar studies in this section suggest that organizations should tailor the acquisition of external knowledge to fit the specific industry and market dynamics. Different search patterns may result in different levels of IP. These results emphasize the importance for companies to thoughtfully consider how to search for and integrate external knowledge into their innovation processes while taking into account their unique capabilities and context (6, 34, 69). Our study is the first of its kind in the biopharmaceutical industry in Iran and is expected to create opportunities for companies to improve their IP.

### 5.2. Investigating the Moderating Effect of R&D Intensity

Our research, along with other studies, suggests that increasing R&D intensity does not always lead to better IP. This aligns with evidence that the quality and strategic alignment of R&D activities may matter more than their scale. For instance, Dong et al. noted a curvilinear relationship between R&D intensity and IP, implying diminishing returns beyond an optimal threshold (70). Similarly, other studies have shown that factors like governance structure (71), commercialization orientation (72), or government subsidies (42) can reshape how R&D translates into innovation conditions that may not fully apply in Iran’s uniformly regulated, resource-constrained environment. Critically, the non-significant moderating role of R&D intensity in our model reflects the structural realities of Iran’s biopharmaceutical sector. Due to technological sanctions, limited access to original cell lines, and intellectual property barriers, firms are largely unable to engage in radical drug discovery. Instead, R&D activities are predominantly oriented toward biosimilar development and process optimization forms of “smart imitation” rather than knowledge-intensive innovation. In such a context, higher R&D spending alone cannot compensate for weak KIC. Without the internal routines to absorb, recombine, and adapt external knowledge, increased budgets risk becoming inefficient or even wasteful. It is also worth noting that the non-significant moderating role of R&D intensity may be partially influenced by social desirability bias, as some managers tended to overreport their R&D expenditures, potentially inflating this metric beyond actual investment levels further weakening its empirical link to innovation outcomes.

### 5.3. Examining the Role of Company Age and Size as Control Variables in Innovation Performance

Our results show that neither firm size nor age significantly affects IP, a finding that challenges conventional assumptions derived largely from other studies, where scale and experience often translate into innovation advantage (73, 74). In contrast, in Iran’s policy-intensive and resource-constrained biopharmaceutical ecosystem, such advantages are decoupled from demographic traits. In this context, many large and established firms are primarily generic drug manufacturers with minimal R&D investment. Their market position stems not from innovation, but from stable government procurement and regulated pricing. Conversely, smaller, and younger biotech firms despite limited scale often demonstrate higher innovation by actively collaborating with universities and strategically sourcing external knowledge.

Thus, organizational size and age lose their typical predictive power. This reflects a broader insight, in policy-driven, resource-constrained ecosystems, strategic choices and external linkages matter more than demographic traits specially in Iran. As Baláž et al. suggest, innovation strategy can mediate the influence of firm age and size (75), while Adam and Alarifi highlight that external support often drives SME innovation under pressure situations (76).

Our null result, therefore, is not a weakness but a contextual signature of Iran’s unique innovation landscape.

### 5.4. Investigating the Mediating Role of Knowledge Integration Capability in the Relationship Between External Knowledge Search Strategy and Innovation Performance

An organization's EKS strategy remains incomplete without the capacity to internally communicate and share insights gained from the external environment. The ability to integrate knowledge includes gathering and processing different information within the organization. In our model, we examined KIC as a mediating variable. This indicates that innovation is driven not only by access to external knowledge but also by a firm's ability to acquire and integrate knowledge into its existing operations and R&D processes. This capability enables firms not only to absorb external knowledge but also to combine it with their internal knowledge, thus enhancing their IP. In this regard, Martini's study also showed that EKS practices have a positive effect on IP. This positive effect becomes more pronounced when internal knowledge management methods are used to integrate and manage innovative ideas. It's also noted that an organization's EKS strategies will not be effective without the ability to communicate and share information internally (77). Dong's study emphasized that KIC forms a vital foundation for digital enterprises. It also highlighted the necessity of a strategic approach to knowledge sharing for enhancing IP within these organizations (78). The positive impact of EKS strategies on KIC is supported by the literature, which highlights the essential role of this variable in enhancing IP. For clarification, we can refer to a study by Feng and his colleagues that emphasizes the importance of dynamic knowledge management capabilities in improving IP. The study demonstrates that effective management and the ability to absorb, transfer, and apply knowledge are crucial. These capabilities are not fixed; they evolve with changes in the business environment, allowing companies to adapt and reconfigure their knowledge resources in response to market conditions, technological advancements, and competitive pressures. This adaptability helps companies to maintain or enhance their IP despite external uncertainties and rapid changes (79). The Trantopoulos study investigates how the acquisition and retention of external knowledge, along with the presence of information technology facilities, collectively impact the process IP of Swiss firms in various manufacturing industries. This research offers valuable insights into how companies can strategically utilize information technology to enhance their innovation capabilities by effectively integrating external knowledge into their processes (80).

We found that biopharmaceutical companies can improve their IP by integrating the acquired knowledge into their operations. This is achieved by holding team meetings that involve experts with different skills, which leads to the generation of new ideas. Sharing information from experts in different units with relevant people in the organization further contributes to increased IP. However, for this to be successful, employees in the company need to have a shared understanding of the processes required to complete projects. Additionally, the company's staff must be well-informed about and comply with the necessary rules, responsibilities, and collaboration measures. Ultimately, these conditions will allow employees to utilize their expertise and knowledge to bring new ideas to fruition.

Our study adds to the knowledge of innovation management in the pharmaceutical industry by emphasizing the significance of external knowledge and its integration with internal knowledge. As companies tackle the challenges of developing new medicines and treatments, it is crucial not to underestimate the strategic value of external knowledge and its fusion with internal resources.

### 5.5. Conclusions

The findings offer an understanding of how EKS enhances IP in Iran’s biopharmaceutical firms. Notably, EKS exerts a strong positive effect on IP (β = 0.462), indicating that firms actively engaging with external sources such as scientific publications, conferences, and collaborative networks achieve substantially higher innovation outcomes. More importantly, KIC emerges as a pivotal mechanism. It not only directly boosts IP (β = 0.437) itself but also partially mediates the EKS-IP link (indirect effect β = 0.104). This implies that about 20% of EKS’s total impact on innovation operates through integration processes, reinforcing that external knowledge alone is insufficient without internal capability to synthesize it.

In contrast, R&D intensity shows no significant moderating role (β = 0.198, P = 0.10), suggesting that higher R&D spending does not amplify the EKS-IP relationship in this context. Furthermore, firm size (β = 0.009) and age (β = 0.03) have negligible effects, confirming that innovation in this emerging ecosystem is driven more by strategic knowledge management than by structural or temporal advantages.

Altogether, these results highlight that KIC is not merely a mediator but a core strategic capability one that biopharmaceutical firms should prioritize to translate external insights into tangible innovation outcomes.

### 5.6. Managerial and Policy Implications

The findings offer actionable guidance for managers and policymakers in Iran’s biopharmaceutical sector.

Managers should strengthen KIC by holding cross-functional team meetings to interpret external knowledge, using shared platforms to document processes and collaboration rules, training staff to combine diverse knowledge into innovations, and assigning knowledge brokers to facilitate information flow across units. Policymakers can support this shift by promoting university–industry R&D consortia, providing innovation vouchers for SMEs to adopt KIC practices, and incentivizing firms that institutionalize structured knowledge integration.

### 5.7. Limitations

- The present study was conducted in the population of biopharmaceutical companies (47 companies), which is considered a small target population.

-Moreover, this population operates under a specific set of institutional, regulatory, and resource constraints. As such, the findings may not be generalizable to firms in other countries, particularly those in developed or differently regulated emerging economies.

- Single research method: Relying only on the questionnaire may limit the scope of the study findings. Different research methods can provide diverse insights, and using only one method may limit the depth and richness of the collected data.

- Response bias: The study uses self-reported measures, and there is a risk of social desirability bias, where respondents may answer in a way, they believe is desirable rather than honestly.

ijpr-25-1-164658-s001.pdf

## Data Availability

All data supporting the findings are included in this article, and the questionnaire as a supplementary file is available.

## References

[1] Weissenberger-Eibl MA, Hampel T (2021). Bridging the gap: integrating external knowledge from open innovation platforms. SN Business & Economics.

[2] Nylund PA, Ferras-Hernandez X, Brem A (2020). Automating profitably together: Is there an impact of open innovation and automation on firm turnover? Review of Managerial Science. 2020 Feb 12;14(1):269–85.

[3] Brunswicker S, Chesbrough H (2018). The Adoption of Open Innovation in Large Firms. Research-Technology Management.

[4] Bogers M, Chesbrough H, Moedas C (2018). Open Innovation: Research, Practices, and Policies. California Management Review.

[5] Zhang Y, Zhang X, Zhang H, A L (2022). The Influence of External Knowledge Searches on Enterprises’ Innovation Performance: A Meta-Analysis. Sustainability.

[6] Kim CY, Lim MS, Yoo JW (2019). Ambidexterity in External Knowledge Search Strategies and Innovation Performance: Mediating Role of Balanced Innovation and Moderating Role of Absorptive Capacity. Sustainability.

[7] Gurgula O (2020). Strategic Patenting by Pharmaceutical Companies – Should Competition Law Intervene? IIC - International Review of Intellectual Property and Competition Law. 2020 Nov 28;51(9):1062–85.

[8] Jangid AK, Agraval H, Gupta N, Yadav UCS, Sistla R, Pooja D (2019). Designing of fatty acid-surfactant conjugate based nanomicelles of morin hydrate for simultaneously enhancing anticancer activity and oral bioavailability. Colloids and Surfaces B: Biointerfaces.

[9] Schuhmacher A, Gassmann O, McCracken N, Hinder M (2018). Open innovation and external sources of innovation. An opportunity to fuel the R&D pipeline and enhance decision making? Journal of Translational Medicine.

[10] Paul SM, Mytelka DS, Dunwiddie CT, Persinger CC, Munos BH, Lindborg SR (2010). How to improve R&D productivity: the pharmaceutical industry’s grand challenge. Nature Reviews Drug Discovery.

[11] Martinez-Grau MA, Alvim-Gaston M (2019). Powered by Open Innovation: Opportunities and Challenges in the Pharma Sector. Pharmaceutical Medicine.

[12] Yuen SSM, Lam HY (2024). Enhancing Competitiveness through Strategic Knowledge Sharing as a Driver of Innovation Capability and Performance. Sustainability.

[13] Palmer M, Chaguturu R (2017). Academia–pharma partnerships for novel drug discovery: essential or nice to have? Expert Opinion on Drug Discovery. 2017 Jun 3;12(6):537–40.

[14] Kim E, Lee I, Kim H, Shin K (2021). Factors Affecting Outbound Open Innovation Performance in Bio-Pharmaceutical Industry-Focus on Out-Licensing Deals. Sustainability.

[15] Dyczkowska J (2020). R&D Narratives in Annual Reports of European Biopharmaceutical Companies. Journal of Innovation and Business Best Practice.

[16] Kogut B, Zander U (1992). Knowledge of the Firm, Combinative Capabilities, and the Replication of Technology. Organization Science.

[17] Grant RM (1996). Toward a knowledge‐based theory of the firm. Strategic Management Journal.

[18] Grant R (2016). Knowledge Management Theories. In: The Palgrave Encyclopedia of Strategic Management. London: Palgrave Macmillan UK.

[19] De LucaLM, Atuahene-Gima K (2007). Market Knowledge Dimensions and Cross-Functional Collaboration: Examining the Different Routes to Product Innovation Performance. Journal of Marketing.

[20] Laursen K, Salter A (2006). Open for innovation: The role of openness in explaining innovation performance among U.K. manufacturing firms. Strategic Management Journal.

[21] Bierly P, Chakrabarti A (1996). Generic knowledge strategies in the U.S. pharmaceutical industry. Strategic Management Journal.

[22] Zhou KZ, Li CB (2012). How knowledge affects radical innovation: Knowledge base, market knowledge acquisition, and internal knowledge sharing. Strategic Management Journal.

[23] Kim YJ, Song S, Sambamurthy V, Lee YL (2012). Entrepreneurship, knowledge integration capability, and firm performance: An empirical study. Information Systems Frontiers.

[24] Wang MC, Chen PC, Fang SC (2018). A critical view of knowledge networks and innovation performance: The mediation role of firms’ knowledge integration capability. Journal of Business Research.

[25] Salunke S, Weerawardena J, McColl-Kennedy JR (2019). The central role of knowledge integration capability in service innovation-based competitive strategy. Industrial Marketing Management.

[26] Li MS, Li J, Li JM, Liu ZW, Deng XT (2023). The Impact of Team Learning Climate on Innovation Performance – Mediating role of knowledge integration capability. Frontiers in Psychology.

[27] Harsono TW, Hidayat K, Iqbal M, Abdillah Y (2024). Creating Sustainable Innovation Performance: A Systematic Review and Bibliometric Analysis. Sustainability.

[28] Yam RCM, Lo W, Tang EPY, Lau AKW (2011). Analysis of sources of innovation, technological innovation capabilities, and performance: An empirical study of Hong Kong manufacturing industries. Research Policy.

[29] Gunday G, Ulusoy G, Kilic K, Alpkan L (2011). Effects of innovation types on firm performance. International Journal of Production Economics.

[30] Ngo LV, O’Cass A (2012). In Search of Innovation and Customer‐related Performance Superiority: The Role of Market Orientation, Marketing Capability, and Innovation Capability Interactions. Journal of Product Innovation Management.

[31] SAMSON D, GLOET M, SINGH P (2017). SYSTEMATIC INNOVATION CAPABILITY: EVIDENCE FROM CASE STUDIES AND A LARGE SURVEY. International Journal of Innovation Management.

[32] Sicotte H, Drouin N, Delerue H (2014). Innovation Portfolio Management as a Subset of Dynamic Capabilities: Measurement and Impact on Innovative Performance. Project Management Journal.

[33] Mendoza-Silva A (2021). Innovation capability: a systematic literature review. European Journal of Innovation Management.

[34] Segarra-Ciprés M, Bou-Llusar JC (2018). External knowledge search for innovation: the role of firms’ innovation strategy and industry context. Journal of Knowledge Management.

[35] Wang C, Chin T, Lin Jheng (2020). Openness and firm innovation performance: the moderating effect of ambidextrous knowledge search strategy. Journal of Knowledge Management.

[36] Sammarra A, Biggiero L (2008). Heterogeneity and Specificity of Inter‐Firm Knowledge Flows in Innovation Networks. Journal of Management Studies.

[37] Guan J, Liu N (2016). Exploitative and exploratory innovations in knowledge network and collaboration network: A patent analysis in the technological field of nano-energy. Research Policy.

[38] Eslami MH, Lakemond N, Brusoni S (2018). The dynamics of knowledge integration in collaborative product development: Evidence from the capital goods industry. Industrial Marketing Management.

[39] Zhang Y, Wang D, Xu L (2021). Knowledge search, knowledge integration and enterprise breakthrough innovation under the characteristics of innovation ecosystem network: The empirical evidence from enterprises in Beijing-Tianjin-Hebei region. PLOS ONE.

[40] Liu B (2021). Matching external search strategies with radical and incremental innovation and the role of knowledge integration capability. Baltic Journal of Management.

[41] Usai A, Fiano F, Messeni PetruzzelliA, Paoloni P, Farina BriamonteM, Orlando B (2021). Unveiling the impact of the adoption of digital technologies on firms’ innovation performance. Journal of Business Research.

[42] Xu J, Wang X, Liu F (2021). Government subsidies, R&D investment and innovation performance: analysis from pharmaceutical sector in China. Technology Analysis & Strategic Management.

[43] Nooteboom B, Van HaverbekeW, Duysters G, Gilsing V, van denOordA (2007). Optimal cognitive distance and absorptive capacity. Research Policy.

[44] Zhu J, Wang Y, Wang C (2019). A comparative study of the effects of different factors on firm technological innovation performance in different high-tech industries. Chinese Management Studies.

[45] Cassiman B, Veugelers R (2006). In Search of Complementarity in Innovation Strategy: Internal R&D and External Knowledge Acquisition. Management Science.

[46] Andries P, Stephan U (2019). Environmental Innovation and Firm Performance: How Firm Size and Motives Matter. Sustainability.

[47] Kijkasiwat P, Phuensane P (2020). Innovation and Firm Performance: The Moderating and Mediating Roles of Firm Size and Small and Medium Enterprise Finance. Journal of Risk and Financial Management.

[48] Coad A, Holm JR, Krafft J, Quatraro F (2018). Firm age and performance. Journal of Evolutionary Economics.

[49] Huergo E, Jaumandreu J (2004). How Does Probability of Innovation Change with Firm Age? Small Business Economics. 2004 Apr;22(3/4):193–207.

[50] Shefer D, Frenkel A (2005). R&D, firm size and innovation: an empirical analysis. Technovation.

[51] Yin C, Salmador MP, Li D, Lloria MB (2022). Green entrepreneurship and SME performance: the moderating effect of firm age. International Entrepreneurship and Management Journal.

[52] Armstrong JS, Overton TS (1977). Estimating Nonresponse Bias in Mail Surveys. Journal of Marketing Research.

[53] Baker WE, Sinkula JM (1999). The Synergistic Effect of Market Orientation and Learning Orientation on Organizational Performance. Journal of the Academy of Marketing Science.

[54] Ritter T, Gemünden HG (2004). The impact of a company’s business strategy on its technological competence, network competence and innovation success. Journal of Business Research.

[55] Kirner E, Kinkel S, Jaeger A (2009). Innovation paths and the innovation performance of low-technology firms—An empirical analysis of German industry. Research Policy.

[56] Zou B, Guo F, Guo J (2019). Antecedents and outcomes of breadth and depth of absorptive capacity: An empirical study. Journal of Management & Organization.

[57] Guo B, Wang Y (2014). Environmental turbulence, absorptive capacity and external knowledge search among Chinese SMEs. Chinese Management Studies.

[58] Kang KH, Kang J (2009). How do firms source external knowledge for innovation? Analysing effects of different knowledge sourcing methods. International Journal of Innovation Management.

[59] Lemańska-Majdzik A (2022). Actions for Knowledge Integration capability in Building an Innovative Enterprise: Organizational Perspective. European Conference on Knowledge Management.

[60] Katila R, Ahuja G (2002). SOMETHING OLD, SOMETHING NEW: A LONGITUDINAL STUDY OF SEARCH BEHAVIOR AND NEW PRODUCT INTRODUCTION. Academy of Management Journal.

[61] Cruz-González J, López-Sáez P, Emilio Navas-López J, Delgado-Verde M (2014). Directions of external knowledge search: investigating their different impact on firm performance in high-technology industries. Journal of Knowledge Management.

[62] Fornell C (1987). A second generation of multivariate analysis: Classification of methods and implications for marketing research. American Marketing Association.

[63] Wynne W (1998). Chin. The Partial Least Squares Approach to Structural Equation Modeling. Psychology Press.

[64] Hair JFetal (2012). The Use of Partial Least Squares Structural Equation Modeling in Strategic Management Research: A Review of Past Practices and Recommendations for Future Applications. Long Range Planning.

[65] Joseph F (2013). Hair, William C. Black, Barry J. Babin REA. Multivariate Data Analysis.

[66] Cheung GW, Cooper-Thomas HD, Lau RS, Wang LC (2024). Reporting reliability, convergent and discriminant validity with structural equation modeling: A review and best-practice recommendations. Asia Pacific Journal of Management.

[67] Fornell C, Larcker DF (1981). Evaluating Structural Equation Models with Unobservable Variables and Measurement Error. Journal of Marketing Research.

[68] Hair J, Hult G, Ringle Cetal (2016). A Primer on Partial Least Squares Structural Equation Modeling (PLS-SEM). SAGE.

[69] Li Z, Zhang K, Dang J, Zheng S, Wang R, Wang Z (2023). Research on the Influence of External Search Strategy on Enterprise Innovation. Ecological Chemistry and Engineering S.

[70] Dong Y, Wei Z, Liu T, Xing X (2020). The Impact of R&D Intensity on the Innovation Performance of Artificial Intelligence Enterprises-Based on the Moderating Effect of Patent Portfolio. Sustainability.

[71] Currim IS, Lim J, Kim JW (2012). You get what you Pay for: The Effect of Top Executives’ Compensation on Advertising and R&D Spending Decisions and Stock Market Return. Journal of Marketing.

[72] Lin BW, Lee Y, Hung SC (2006). R&D intensity and commercialization orientation effects on financial performance. Journal of Business Research.

[73] Zhou L (2007). The effects of entrepreneurial proclivity and foreign market knowledge on early internationalization. Journal of World Business.

[74] Jiménez-Jiménez D, Sanz-Valle R (2011). Innovation, organizational learning, and performance. Journal of Business Research.

[75] Baláž V, Jeck T, Balog M (2023). Firm performance over innovation cycle: evidence from a small European economy. Journal of Innovation and Entrepreneurship.

[76] Adam NA, Alarifi G (2021). Innovation practices for survival of small and medium enterprises (SMEs) in the COVID-19 times: the role of external support. Journal of Innovation and Entrepreneurship.

[77] Martini A, Neirotti P, Appio FP (2015). Knowledge Searching, Integrating and Performing: Always a Tuned Trio for Innovation? Long Range Planning. 2015;50(2):200–20.

[78] Dong H, Guo J, Chen T, Murong R (2023). Configuration research on innovation performance of digital enterprises: Based on an open innovation and knowledge perspective. Frontiers in Environmental Science.

[79] Feng L, Zhao Z, Wang J, Zhang K (2022). The Impact of Knowledge Management Capabilities on Innovation Performance from Dynamic Capabilities Perspective: Moderating the Role of Environmental Dynamism. Sustainability.

[80] Trantopoulos K, von KroghG, Wallin MW, Woerter M (2017). External Knowledge and Information Technology: Implications for Process Innovation Performance. MIS Quarterly.

