# Supplementary material for: External Knowledge Search and Innovation Performance in Iranian Biopharmaceutical Firms: The Mediating Role of Knowledge Integration Capability
Source: Iran J Pharm Res. 2026 May 15;25(1):e164658. doi: 10.5812/ijpr-164658 (PMC13389372; doi:10.5812/ijpr-164658)
Supplement: ijpr-25-1-164658-s001.pdf [file ijpr-25-1-164658-s001.pdf]

Shahid Beheshti University of Medical Sciences
School of Pharmacy

Dear Participant,

This research, titled "External Knowledge Search and Innovation Performance in Iranian Biopharmaceutical Firms: The Mediating Role of Knowledge Integration Capability" aims to examine how externally acquired knowledge influences innovation performance specifically, the successful development and market entry of new products.

In this regard, we kindly request your valuable participation by completing this questionnaire. Please select the option that best reflects the current situation of your company.

All information and responses provided will be treated with strict confidentiality and used solely for academic research purposes.

Thank you very much for your time and support.

Sincerely,
The Research Team

**Company Information**<br><br>Company Name: --------------- Year of Establishment: --------------<br>Total Number of Employees in the Company: --------------<br>Number of Employees in the R&D Department: --------------<br><br>**Your Position in the Company:**<br><br>CEO / Managing Director ☐<br>Senior R&D Manager ☐<br>Senior Business Development Manager ☐<br>R&D Manager ☐<br>Business Development Manager ☐
**Your tenure at the company:** _____ years<br>**In the past five years, in which of the following areas has your company carried out innovative projects or activities?**<br>(Please check all that apply)<br>Improvement or enhancement of existing products ☐<br>Development of products similar to those of competitors ☐<br>New and innovative products introduced to the market ☐

**Questions Related to the Breadth and Depth of External Knowledge Search**<br>Breadth of external knowledge search refers to the *number of external sources or channels* a firm uses to acquire knowledge.<br>Depth of external knowledge search refers to the *extent to which the firm actively engages with and leverages* those diverse external knowledge sources.		
Please answer the following questions based on successful new products that your company has commercialized in the past five years, and in which you played a role in their development.<br>***Has your company used any of the external information sources listed below to generate ideas for new innovations or to support ongoing innovative projects?**<br>(After indicating "Yes" or "No," please rate the extent of use by marking the appropriate box for each external source.)		
**Questions**	**Responses**	**Notes**
1. Suppliers of equipment, materials, components, or software	Yes ☐ No ☐<br>Very low ☐ Low ☐ Medium ☐ High ☐ Very high ☐	
2. Customers	Yes ☐ No ☐<br>Very low ☐ Low ☐ Medium ☐ High ☐ Very high ☐	

3. Competitors	Yes ☐ No ☐<br>Very low ☐ Low ☐ Medium ☐ High ☐ Very high ☐	
4. Business consultants	Yes ☐ No ☐<br>Very low ☐ Low ☐ Medium ☐ High ☐ Very high ☐	
5. Informal employee interactions with employees of other companies	Yes ☐ No ☐<br>Very low ☐ Low ☐ Medium ☐ High ☐ Very high ☐	
6. Hiring experienced employee from other companies	Yes ☐ No ☐<br>Very low ☐ Low ☐ Medium ☐ High ☐ Very high ☐	
7. Consulting contracts with external parties for technical knowledge transfer or workforce training	Yes ☐ No ☐<br>Very low ☐ Low ☐ Medium ☐ High ☐ Very high ☐	
8. Exhibitions	Yes ☐ No ☐<br>Very low ☐ Low ☐ Medium ☐ High ☐ Very high ☐	
9. Patents	Yes ☐ No ☐<br>Very low ☐ Low ☐ Medium ☐ High ☐ Very high ☐	
10. Scientific journals	Yes ☐ No ☐<br>Very low ☐ Low ☐ Medium ☐ High ☐ Very high ☐	
11. Press or technical and commercial sites	Yes ☐ No ☐<br>Very low ☐ Low ☐ Medium ☐ High ☐ Very high ☐	
12. Specialized and international conferences	Yes ☐ No ☐<br>Very low ☐ Low ☐ Medium ☐ High ☐ Very high ☐	
13. National meetings and congresses	Yes ☐ No ☐<br>Very low ☐ Low ☐ Medium ☐ High ☐ Very high ☐	
14. Business associations (e.g., chambers of commerce)	Yes ☐ No ☐<br>Very low ☐ Low ☐ Medium ☐ High ☐ Very high ☐	
15. Commercial laboratories / R&D companies	Yes ☐ No ☐<br>Very low ☐ Low ☐ Medium ☐ High ☐ Very high ☐	
16. Universities or other higher education institutions	Yes ☐ No ☐<br>Very low ☐ Low ☐ Medium ☐ High ☐ Very high ☐	
17. Government or private research organizations	Yes ☐ No ☐<br>Very low ☐ Low ☐ Medium ☐ High ☐ Very high ☐	
**Questions Related to Knowledge Integration Capability (KIC)**<br>Knowledge Integration Capability refers to the set of activities undertaken to combine broad and dispersed external knowledge with a firm's internal knowledge through intra-organizational interactions and collective learning.		
Please answer the following questions, taking into account your company's internal conditions and intra-organizational knowledge interactions in the context of carrying out innovative projects.		
**Questions**	**Responses**	**Notes**
1. Our company uses existing organizational knowledge to develop new products or services.	Strongly disagree ☐ Disagree ☐ Somewhat disagree ☐ Neutral ☐ Somewhat agree ☐ Agree ☐ Strongly agree ☐	
2. Our company can receive external knowledge in a timely manner.	Strongly disagree ☐ Disagree ☐ Somewhat disagree ☐ Neutral ☐ Somewhat agree ☐ Agree ☐ Strongly agree ☐	

3. Our company is able to combine external and internal knowledge and apply it in new domains.	Strongly disagree ☐ Disagree ☐ Somewhat disagree ☐ Neutral ☐ Somewhat agree ☐ Agree ☐ Strongly agree ☐	
4. Our company can integrate the acquired EK into organizational performance.	Strongly disagree ☐ Disagree ☐ Somewhat disagree ☐ Neutral ☐ Somewhat agree ☐ Agree ☐ Strongly agree ☐	
5. Various types of knowledge and information sources acquired by our company are systematically documented.	Strongly disagree ☐ Disagree ☐ Somewhat disagree ☐ Neutral ☐ Somewhat agree ☐ Agree ☐ Strongly agree ☐	
6. In our company, team meetings involve experts with different skills to generate new ideas.	Strongly disagree ☐ Disagree ☐ Somewhat disagree ☐ Neutral ☐ Somewhat agree ☐ Agree ☐ Strongly agree ☐	
7. Our company shares information from experts in different units with relevant people in the organization.	Strongly disagree ☐ Disagree ☐ Somewhat disagree ☐ Neutral ☐ Somewhat agree ☐ Agree ☐ Strongly agree ☐	
8. Our company uses past experiences to refine resource allocation methods, especially for accurately allocating scarce resources.	Strongly disagree ☐ Disagree ☐ Somewhat disagree ☐ Neutral ☐ Somewhat agree ☐ Agree ☐ Strongly agree ☐	
9. Employees in the company have a shared understanding of the processes required to complete projects.	Strongly disagree ☐ Disagree ☐ Somewhat disagree ☐ Neutral ☐ Somewhat agree ☐ Agree ☐ Strongly agree ☐	.
10. The company's staff is well-informed about and complies with the necessary rules, responsibilities, and collaboration measures.	Strongly disagree ☐ Disagree ☐ Somewhat disagree ☐ Neutral ☐ Somewhat agree ☐ Agree ☐ Strongly agree ☐	
11. All employees in the company can use their expertise and knowledge to bring new ideas to fruition.	Strongly disagree ☐ Disagree ☐ Somewhat disagree ☐ Neutral ☐ Somewhat agree ☐ Agree ☐ Strongly agree ☐	
12. Individual employees in our company can gain mastery over external knowledge entering the organization.	Strongly disagree ☐ Disagree ☐ Somewhat disagree ☐ Neutral ☐ Somewhat agree ☐ Agree ☐ Strongly agree ☐	
13. Our company provides employees with opportunities to learn from external knowledge sources.	Strongly disagree ☐ Disagree ☐ Somewhat disagree ☐ Neutral ☐ Somewhat agree ☐ Agree ☐ Strongly agree ☐	
14. Employees in our company can integrate knowledge acquired from outside the organization with their existing knowledge.	Strongly disagree ☐ Disagree ☐ Somewhat disagree ☐ Neutral ☐ Somewhat agree ☐ Agree ☐ Strongly agree ☐	
15. Employees in our company are able to apply externally acquired knowledge in new projects.	Strongly disagree ☐ Disagree ☐ Somewhat disagree ☐ Neutral ☐ Somewhat agree ☐ Agree ☐ Strongly agree ☐	

**Questions Related to Innovation Performance**<br>This section examines the quality, efficiency, and overall performance of the company's innovation activities.		
Please answer the following questions, considering either the successful new products your company has brought to market in the past five years, or your organization's overall conditions.		
**Questions**	**Responses**	**Notes**
1. The number of our products is increasing.	Strongly disagree ☐ Disagree ☐ Somewhat disagree ☐ Neutral ☐ Somewhat agree ☐ Agree ☐ Strongly agree ☐	
2. The share of sales revenue from our new products, compared to total sales, is increasing.	Strongly disagree ☐ Disagree ☐ Somewhat disagree ☐ Neutral ☐ Somewhat agree ☐ Agree ☐ Strongly agree ☐	
3. Our company is seeing an increase in the introduction of new products.	Strongly disagree ☐ Disagree ☐ Somewhat disagree ☐ Neutral ☐ Somewhat agree ☐ Agree ☐ Strongly agree ☐	
4. We have improved the efficiency of delivering our products to market	Strongly disagree ☐ Disagree ☐ Somewhat disagree ☐ Neutral ☐ Somewhat agree ☐ Agree ☐ Strongly agree ☐	
5. Our new product development program has met its set objectives.	Strongly disagree ☐ Disagree ☐ Somewhat disagree ☐ Neutral ☐ Somewhat agree ☐ Agree ☐ Strongly agree ☐	
6. Most of our new product development programs are profitable.	Strongly disagree ☐ Disagree ☐ Somewhat disagree ☐ Neutral ☐ Somewhat agree ☐ Agree ☐ Strongly agree ☐	
7. Our new products clearly offer more advantages than the previous ones.	Strongly disagree ☐ Disagree ☐ Somewhat disagree ☐ Neutral ☐ Somewhat agree ☐ Agree ☐ Strongly agree ☐	
8. Our overall new product development program is much more successful compared to that of our main competitors.	Strongly disagree ☐ Disagree ☐ Somewhat disagree ☐ Neutral ☐ Somewhat agree ☐ Agree ☐ Strongly agree ☐	
9. We always release new products before our competitors	Strongly disagree ☐ Disagree ☐ Somewhat disagree ☐ Neutral ☐ Somewhat agree ☐ Agree ☐ Strongly agree ☐	
10. Our new products are gaining more market share.	Strongly disagree ☐ Disagree ☐ Somewhat disagree ☐ Neutral ☐ Somewhat agree ☐ Agree ☐ Strongly agree ☐	
11. Our new products create new markets for themselves.	Strongly disagree ☐ Disagree ☐ Somewhat disagree ☐ Neutral ☐ Somewhat agree ☐ Agree ☐ Strongly agree ☐	
12. After our new products entered the market, they achieved remarkable success in terms of sales.	Strongly disagree ☐ Disagree ☐ Somewhat disagree ☐ Neutral ☐ Somewhat agree ☐ Agree ☐ Strongly agree ☐	
13. The market responds positively to our new product development initiatives.	Strongly disagree ☐ Disagree ☐ Somewhat disagree ☐ Neutral ☐ Somewhat agree ☐ Agree ☐ Strongly agree ☐	
**Question Related to Firm R&D Intensity**		
Please answer the following question, taking into account your organization's overall conditions.		

Questions	Responses	Notes
What percentage of your company's total sales revenue is allocated to R&D expenditures?	Less than 5% ☐<br>5–15% ☐<br>15–25% ☐<br>25–35% ☐<br>More than 35% ☐	
